# Neutrophil infiltration in peritoneal metastasis affects prognosis in patients with ovarian cancer

**DOI:** 10.1038/s41598-025-05010-3

**Published:** 2025-07-02

**Authors:** Emiri Miyamoto, Masato Yoshihara, Shohei Iyoshi, Kazumasa Mogi, Kaname Uno, Hiroki Fujimoto, Yoshihiro Koya, Kazuhisa Kitami, Kosuke Yoshida, Satoshi Tamauchi, Akira Yokoi, Nobuhisa Yoshikawa, Kaoru Niimi, Hiroyuki Tomita, Hiroyasu Kidoya, Yukihiro Shiraki, Atsushi Enomoto, Hiroaki Kajiyama

**Affiliations:** 1 Department of Obstetrics and Gynecology, Nagoya University Graduate School of Medicine, 65, Tsuruma-cho, Showa-ku, Nagoya, Aichi Japan; 2 Institute for Advanced Research, Nagoya University, Nagoya, Aichi Japan; 3 Department of Medical Genomics Center, Nagoya University, Nagoya, Aichi Japan; 4 Division of Oncology, Department of Clinical Sciences, Lund University, Lund, Sweden; 5 Department of Gynecologic Oncology, Aichi Cancer Center, Nagoya, Aichi Japan; 6 Department of Tumor Pathology, Graduate School of Medicine, Gifu University, Gifu, Japan; 7 Department of Integrative Vascular Biology, Faculty of Medical Sciences, University of Fukui, Fukui, Japan; 8 Department of Pathology, Graduate School of Medicine, Nagoya University, Nagoya, Aichi Japan

**Keywords:** Ovarian carcinoma, Neutrophil, Prognosis, Peritoneal metastasis, Cancer, Oncology

## Abstract

The impact on prognosis of neutrophil infiltration in the microenvironment of ovarian cancer remains to be elucidated. In this study, we analyzed the association between neutrophil infiltration in peritoneal metastasis and prognosis. Furthermore, we analyzed the correlation between neutrophil infiltration in peritoneal metastasis and the number of peripheral blood neutrophils, the degree of neutropenia, vascular endothelial area, and the number of stromal cells. Thirty-four specimens each of primary and metastatic advanced ovarian cancer with high-grade serous carcinoma histology were taken. Staining for MPO, CD31, and αSMA was performed on all specimens. We investigated the correlation among tissue neutrophil infiltration, prognosis, and neutrophil indicators from blood tests. Survival analysis showed that neutrophil infiltration in the primary tumor had no impact on prognosis, whereas high neutrophil infiltration in the disseminated tumor significantly shortened progression-free survival and overall survival. No correlation was found between neutrophil infiltration and the number of peripheral blood neutrophils, the degree of neutropenia, area of vascular endothelial cells, and number of stromal cells. Neutrophil infiltration into disseminated lesions of the omentum has a significant impact on prognosis. The results of the present study may provide insights for refining clinical approaches.

**Supplementary Information:**

The online version contains supplementary material available at 10.1038/s41598-025-05010-3.

## Introduction

Ovarian cancer has the poorest prognosis of all gynecological malignancies, causing peritoneal dissemination from an early stage^1^. Despite good initial response to treatment, advanced cancers have a high rate of recurrence and a very low survival rate after recurrence^2,3^. Peritoneal dissemination recurrence occurs in most cases and an effective treatment remains to be elucidated. As the tumor burden of peritoneal dissemination is extremely high and eradication at the cellular level is not possible, targeted therapy aimed at disease control rather than radical cure is desirable^4^. Unlike primary tumors, peritoneally disseminated tumors are accompanied by an increase in tumor stroma, and it has been suggested that this characteristic tumor microenvironment may be the cause of resistance to treatment^5–7^. Thus, elucidating the microenvironment of peritoneally disseminated tumors has important implications for improving therapeutic targeting.

Neutrophils are the most abundant cells in the human leukemic fraction, accounting for around 60–70%^8^. They differentiate in the bone marrow into mature neutrophils via hematopoietic stem cells, progenitor cells, and myeloblasts^9^. They are induced by infection and play a crucial role in the initial phases of the immune response^10^. Neutrophils have several functions in the cancer microenvironment, including cancer-suppressive functions, such as direct cytotoxicity^11–13^and cancer-promoting functions such as immune escape^14,15^ and contributing to angiogenesis^16–18^.

In ovarian cancer, there are many reports of the prognostic relevance of neutrophils. Previous studies have reported that a high neutrophil-lymphocyte ratio (NLR) in ovarian cancer is associated with poor prognosis^19–21^. Neutrophil extracellular traps (NETs) released by neutrophils promote hematogenous metastasis of ovarian cancer^22^and elevated NETosis markers in peripheral blood are linked to poor prognosis^23^. On the other hand, greater neutropenia during chemotherapy is associated with an improved prognosis^24^. The relationship between neutrophils in the peritoneally disseminated cancer microenvironment of ovarian cancer and life expectancy and neutrophils in the peripheral blood remains to be elucidated.

In this study, we analyzed the association between neutrophil infiltration in peritoneal metastasis and prognosis. Furthermore, the correlation between neutrophil infiltration in peritoneal metastasis and the number of peripheral blood neutrophils, the degree of neutropenia, vascular endothelial area, and the number of stromal cells was also analyzed.

## Patients and methods

### Study participants

We registered patients with malignant ovarian tumors treated at Nagoya University Hospital between September 2004 and November 2018. The present study was approved by the Nagoya University Hospital ethics committee and was conducted in accordance with the principles of the Declaration of Helsinki. The ethical approval number is 2020 − 0570. Data were collected from medical records and clinical follow-up visits; therefore, written informed consent was waived for some participants. Since this study did not involve any interventions, an opt-out procedure was implemented as a substitute for informed consent.

Inclusion criteria consisted of patients with advanced ovarian cancer of the high-grade serous carcinoma histological type, who underwent initial surgery, had available histopathological specimens of both primary and disseminated tumors, and had sufficient blood test and chemotherapy data. We included 34 patients and 68 histopathological specimens of primary and disseminated lesions taken from each patient. All patients background is shown in Table 1. Included patients ranged from 38 to 74 years of age, with a median age of 55 years. All patients had received 4–10 courses of standard platinum-based chemotherapy, with 2 patients receiving Bevacizumab. Neoadjuvant chemotherapy (NAC) was performed in 8 patients.

**Table 1:** Baseline data on the 34 patients including high and low neutrophil infiltration groups.

		All	Neutrophil infiltration low	Neutrophil infiltration high	*P*-value
Age		38–74 (55)	43–71 (56.67)	38–74 (53.75)	0.892
Stage	IIIA	0	0	0	0.222
	IIIB	4	1	3	
	IIIC	24	13	11	
	IVA	3	3	0	
	IVB	3	1	2	
pT	3a	1	0	1	0.252
	3b	4	1	3	
	3c	29	17	12	
CA125		59.3-33193 (3446.7)	59–33,193 (4239.711)	136–10,959 (2554.525)	0.187
Residual tumor	complete	6	3	3	0.984
	optimal	11	6	5	
	suboptimal	17	9	8	
NAC	Not performed	26	12	14	0.153
	Performed	8	6	2	

Preoperative blood test data was measured within a month before surgery. The degree of neutropenia was defined using the values after initial chemotherapy and at the lowest point during chemotherapy. Three indices were evaluated: neutrophil count, the amount of neutrophil decline compared with the pre-chemotherapy value, and the rate of decline.

Histopathological slides were reviewed by an expert pathologist according to the criteria of the World Health Organization classification^25^ who had no knowledge of the clinical data of the patients. Sufficient data were available on survival outcomes. Clinical staging was performed by the system established by the International Federation of Gynecology and Obstetrics^26^.

### Surgery, chemotherapy, and follow-up

Patients underwent primary surgery that primarily consisted of hysterectomy and bilateral salpingo-oophorectomy, as well as a comprehensive peritoneal evaluation, including aspiration or wash cytology, biopsy and/or omentectomy, staging lymphadenectomy, and peritoneal exploration. In some patients, incomplete surgery, including uterine-preserved surgery and the omission of staging lymphadenectomy, was performed for clinical reasons such as advanced disease. Details on adjuvant chemotherapy in each period were described in our previous study^27^. Patients were followed up every 1–3 months in the first and second years, every 3–6 months in the third to fifth years, and annually until ten years using a gynecological examination with a CA125 evaluation and ultrasonography as well as periodic radiologic imaging with computed tomography, magnetic resonance imaging, and/or positron emission tomography. Recurrence was diagnosed based on radiological and/or clinical findings. Progression-free survival (PFS) was defined as the time interval between the date of treatment started to that of recurrence or the last follow-up visit. Overall survival (OS) was defined as the time interval between the date of the initial surgery to that of death or the last follow-up visit.

### Human tissues

All studies using human tissues were reviewed by the Ethics Committee of Nagoya University and were conducted in accordance with the principles of the Declaration of Helsinki. This study only used residual tissues that were delinked from identifiers. Formalin-fixed paraffin-embedded (FFPE) sections of primary tumor and omental tissues of women with a confirmed diagnosis of FIGO stage III or IV HGSC were selected.

### Immunohistochemical analysis

FFPE ovarian cancer tissues were cut into 4 μm thick sections. Utilizing rabbit polyclonal anti-MPO antibody, we aimed to identify and quantify the expression of MPO molecules within the tissues. We also used mouse monoclonal anti-CD31 and anti-αSMA antibody. Sources of antibody for the indicated applications for immunohistochemistry were MPO (Dako, A0398, 1:200), CD31 (Novus, NBP2-44339, 1A10, 1:200), and αSMA (abcam, ab7817, 1A4, 1:200). Slides for analysis were prepared with the Olympus Corporation VS120 slide scanner. We evaluated the number or the rate of MPO-positive cell per unit field of view with HALO (Indica Labs) with the Multiplex IHC Module. Similarly, we evaluated CD31-positive area and the rate of αSMA-positive cell per unit field of view. All analysis was calculated in three randomly selected fields in each sample. Negative controls were run on all sections in blocking buffer generated against unrelated antigens.

### Statistical analysis

Kaplan-Meier curves were generated to compare PFS and OS rates among the following groups: high vs. low MPO-positive cells, high vs. low neutrophil counts in peripheral blood tests, more vs. less neutropenia, more vs. fewer vascular endothelial cells, and more vs. fewer stromal cells. Log-rank test was used to identify differences in survival between the groups being compared. Shapiro-Wilk test was performed to confirm the distribution of normality. Comparisons between groups were performed using Student’s *t*-test for continuous variables and chi-square or Fisher’s exact test for categorical variables. Cox regression analysis was also performed to evaluate each predictor of PFS and OS. Pearson correlation coefficient was calculated to examine the correlation between neutrophil infiltration in disseminated lesions and neutrophils in peripheral blood testing, neutropenia, area of vascular endothelium, and number of stromal cells. Significance was set as two-sided with a P value < 0.05. All statistical analyses were conducted using IBM SPSS Statistics, Version 28.0 (IBM Corp., Armonk, NY, USA).

## Results

1. Neutrophil infiltration in primary tumor and peritoneal metastasis.

**Fig. 1:**
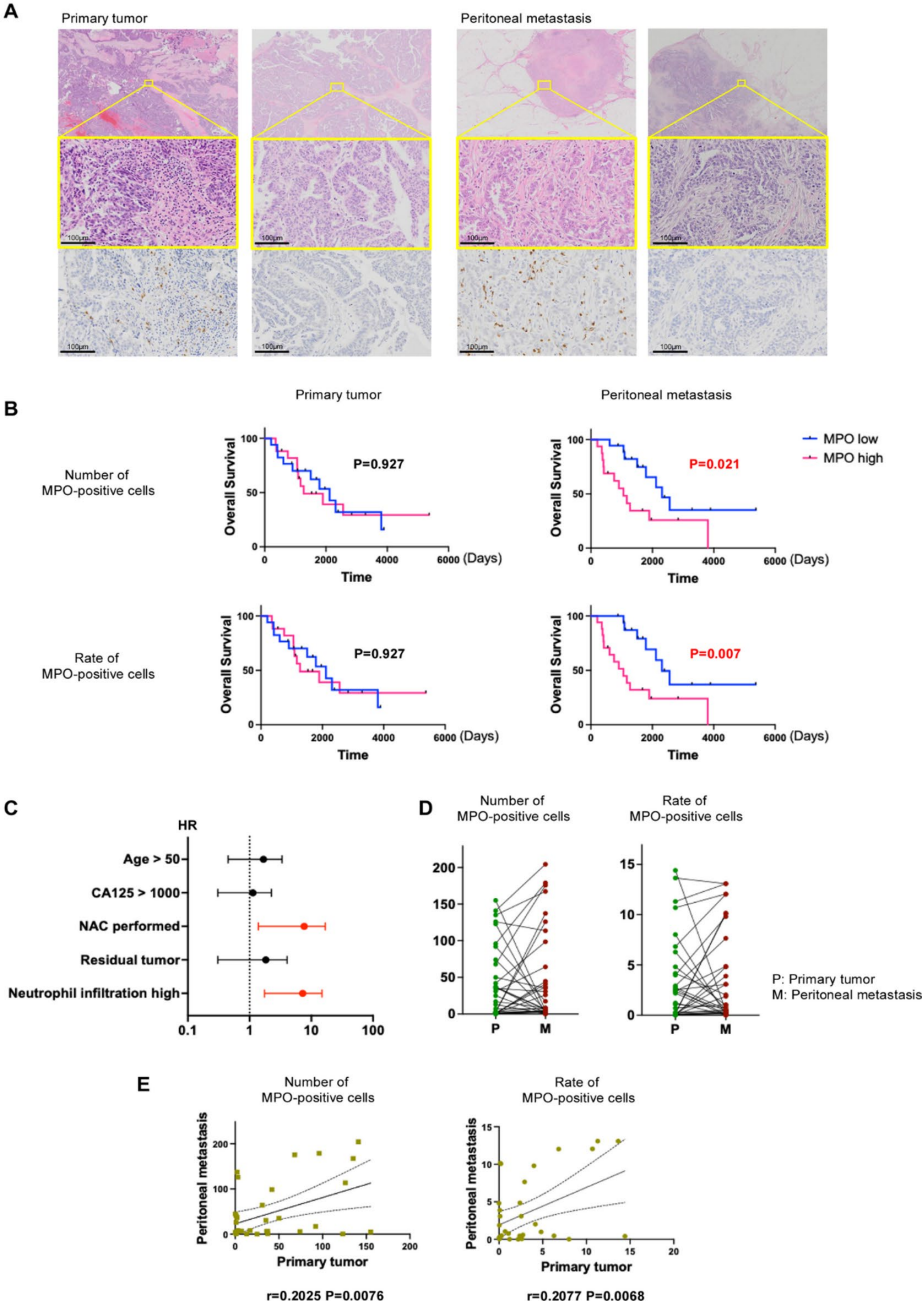
(**A**) HE staining and MPO staining images. High and low neutrophil infiltration in primary tumor or peritoneal metastasis are shown. (**B**) Kaplan–Meier curves are shown for overall survival in high and low neutrophil infiltration groups. (**C**) Forest plot of multivariate analysis for overall survival. (**D**) Before-after graph of the number of MPO-positive cells and rate of MPO-positive cells. Lines connect the value in the primary tumor with that in peritoneal metastasis. (**E**) Correlation between number or rate of MPO-positive cells in primary tumor and that in peritoneal metastasis.

After MPO staining was performed in the 68 specimens, we found that the number of MPO-positive cells varied in both the primary and disseminated tumors (Fig. 1A and Supplementary Fig. 1A). Image analysis was performed using HALO to determine the number of MPO-positive cells per unit area and the MPO-positive cell rate. The median number of MPO-positive cells per unit area was calculated and divided into two groups: high and low neutrophil infiltration in the primary tumor, and high and low neutrophil infiltration in the disseminated tumor. Survival analysis showed that neutrophil infiltration in the primary tumor had no impact on prognosis, whereas high neutrophil infiltration in the disseminated tumor significantly shortened PFS and OS (Fig. 1B and Supplementary Fig. 1A). A similar analysis using the rate of MPO-positive cells per unit area yielded similar results (Fig. 1B and Supplementary Fig. 1A). We compared background factors in the groups with high and low neutrophil infiltration in disseminated lesions to check for confounding factors. We found no significant differences in patient background (Table 1). Furthermore, multivariate analysis revealed that, in addition to NAC, high neutrophil infiltration in disseminated lesions was a significant prognostic factor (Fig. 1C and Supplementary Fig. 1B). There was no correlation between the number or the rate of MPO-positive cells in the primary and disseminated tumors (Fig. 1D and E).

2. Neutrophil indicators in peripheral blood.

**Fig. 2:**
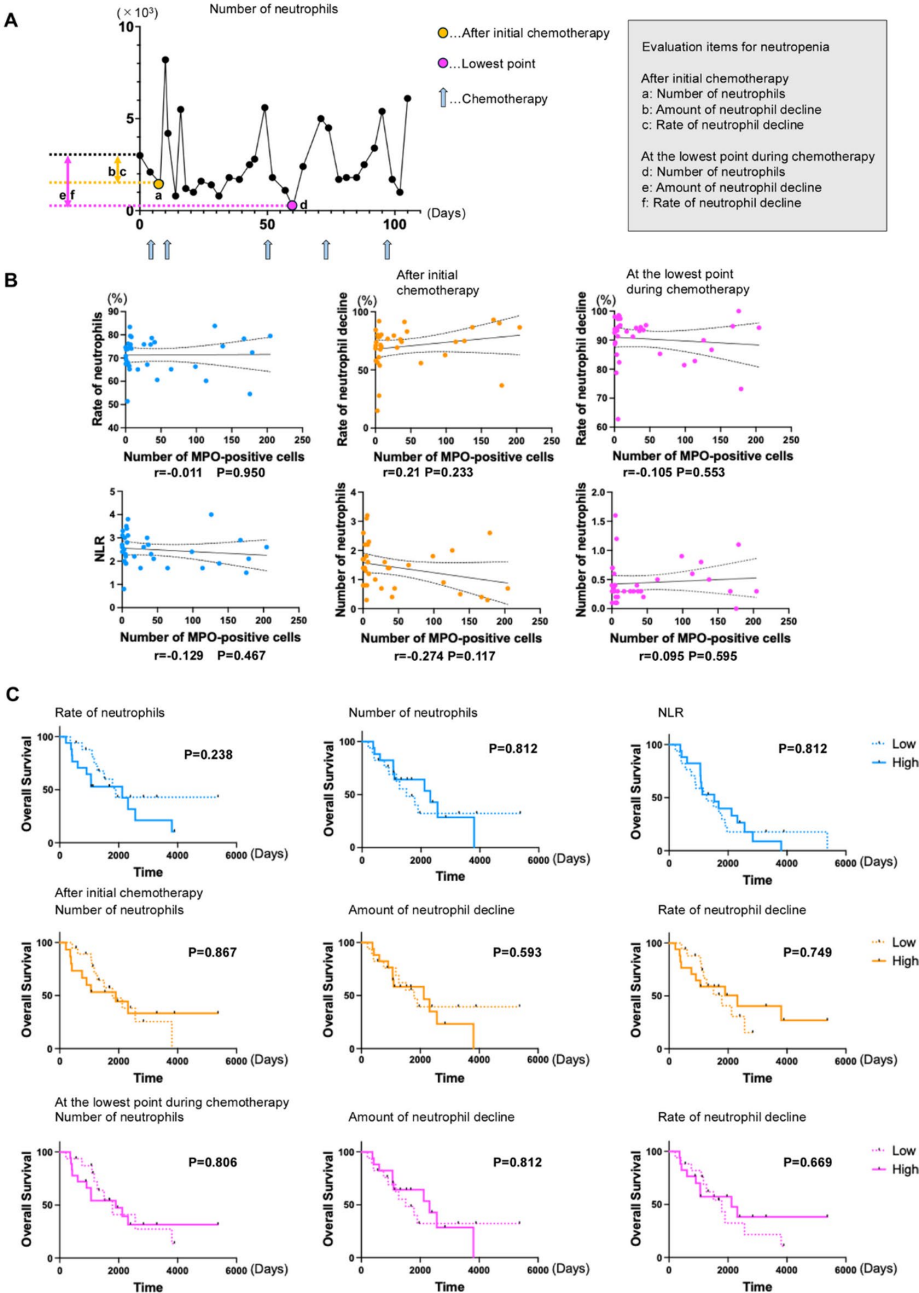
(**A**) The number of neutrophil transitions in a case during chemotherapy and the definitions of evaluation items for neutropenia. (**B**) Correlation between the number of MPO-positive cells and each neutrophil indicator or evaluation item for neutropenia. r: Pearson’s correlation coefficient. (**C**) Kaplan–Meier curves are shown for overall survival in each neutrophil indicator or evaluation item for neutropenia.

As it has been reported that neutropenia during chemotherapy and NLR are associated with prognosis, we examined whether they are associated with neutrophil infiltration of the disseminated tumor. The degree of neutropenia was determined after initial chemotherapy and at the lowest point during chemotherapy, with three indices for each time point: neutrophil count, the amount of neutrophil decline compared with the pre-chemotherapy value, and the rate of decline (Fig. 2A). There was no correlation between disseminated lesion neutrophil infiltration and either the numbers of neutrophils in the peripheral blood or the degree of neutropenia (Fig. 2B and Supplementary Fig. 2A). In addition, the neutrophil index in the peripheral blood and the degree of neutropenia did not have a significant impact on prognosis (Fig. 2C and Supplementary Fig. 2B).

3. Associations of neutrophils and other factors in tumor microenvironment.

**Fig. 3:**
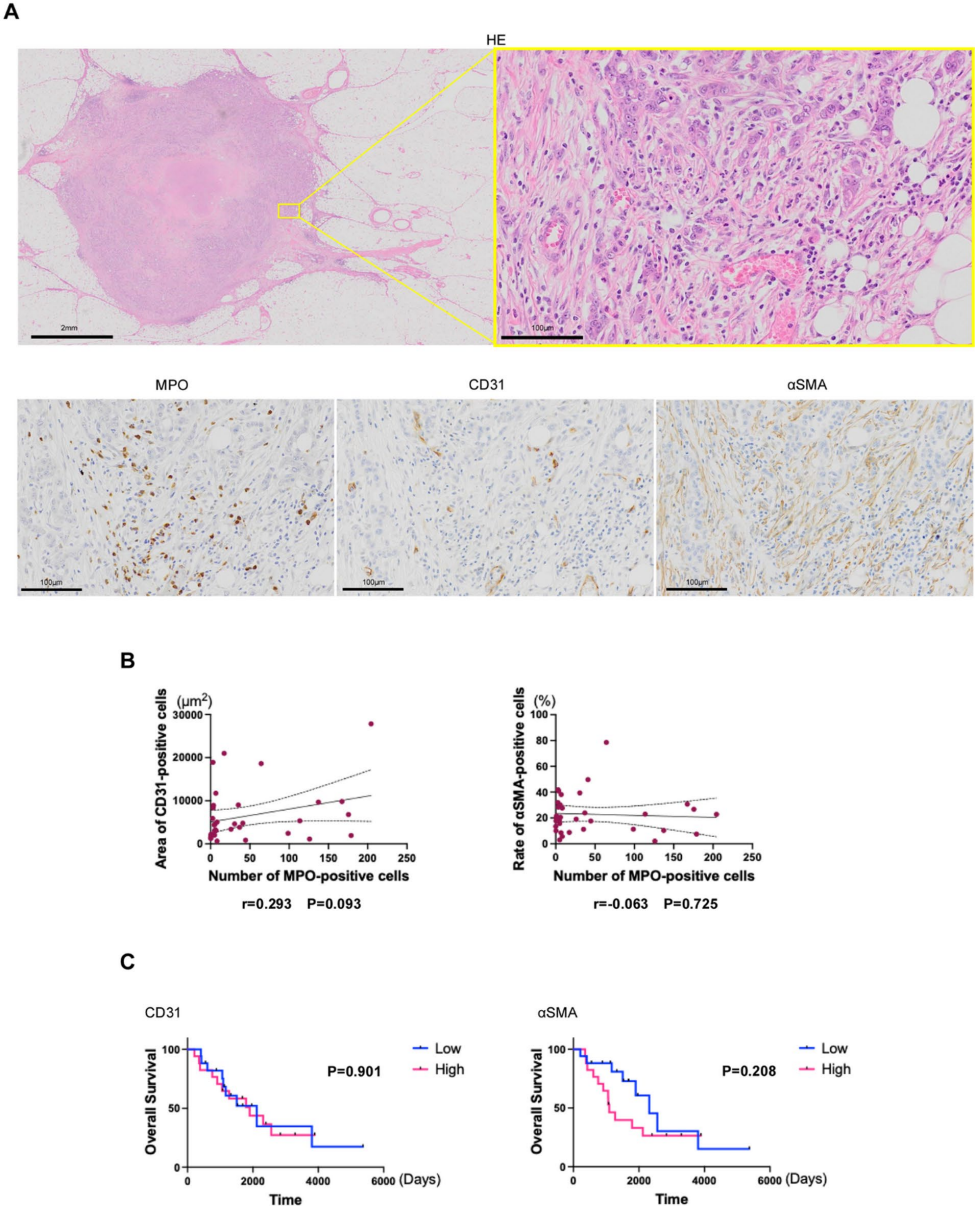
(**A**) HE staining, MPO staining, CD31 staining, and αSMA staining images in peritoneal metastasis. (**B**) Correlation between number of MPO-positive cells and vascular endothelial cell area or number of stromal cells. r: Pearson’s correlation coefficient. (**C**) Kaplan–Meier curves are shown for overall survival in vascular endothelial cell area or the number of stromal cells in peritoneal metastasis.

Finally, we investigated the relationship between neutrophil infiltration, blood vessels, and stroma in disseminated lesions since they are reported to affect neutrophil infiltration. We performed immunohistochemistry of CD31, a marker of vascular endothelial cells, and αSMA, a marker of stromal cells (Fig. 3A). We found no significant correlation between neutrophil infiltration in disseminated lesions and vascular endothelial cell area or the number of stromal cells (Fig. 3B). Shorter OS was found in the group with a larger number of stromal cells, but it was not significant. The vascular endothelial area at the disseminated lesion did not affect prognosis (Fig. 3C and Supplementary Fig. 3).

## Discussion

Our findings suggest that the degree of neutrophil infiltration in peritoneal disseminated lesions is a prognostic factor in advanced ovarian cancer. Although no significant association between peripheral blood neutrophils and histological findings was evident, a mechanism may exist that promotes the progression of ovarian cancer.

There are several previous studies that reported that neutrophil infiltration in tissues promotes cancer progression. Lee et al. reported that NET formation in the omentum promotes metastasis of ovarian cancer^22^. Furthermore, in vivo analyses reported that neutrophils were already upregulated in the omentum during tumor formation in the primary tumor and in early human ovarian cancer specimens without metastatic disease in the omentum^28–30^. Deng et al. reported that neutrophil recruitment to tumor tissue promoted tumor metastasis^28^. These studies suggest that neutrophil infiltration of premetastatic niches is involved in seeding formation. The present results may also be the result of neutrophil infiltration promoting dissemination formation.

There are reports that suppressing neutrophil infiltration suppresses tumor formation; thus, suppressing neutrophil infiltration may be a therapeutic target. There are several reports demonstrating the diversity of neutrophils; however, definitions differ between reports, and it is assumed that cancer-associated neutrophils are not a homogeneous population, but a diverse fraction^31–34^. For example, there are reports that STAT3 activation alters neutrophil phenotype^29^ and that neutrophil functions such as NETs^22^ and ferroptosis^30^ contribute to cancer promotion. These studies suggest that suppression of specific phenotypes or functions of neutrophils may be effective to be applied to ovarian cancer treatment in the future, but further accumulation of knowledge through basic research is needed. The results of the present study bridge the fields of basic, translational, and applied research.

There are studies that reported NLR being associated with prognosis in ovarian cancer^19–21^. Some of these studies included meta-analyses, which had different case numbers compared with the present study. As our study had fewer case numbers, this may explain why no significant differences were found. With regard to neutropenia, the number of cases with NAC in this study was small (eight cases); therefore, it was not possible to make comparisons only among cases with NAC. Further studies with more cases are necessary.

We found no significant association between vascular area and stroma and neutrophil infiltration in the tissue. Previous studies reported that neutrophils induced angiogenesis, that more immune cells were observed in tissues with loose fibronectin and collagen, and that immune cell abundance was correlated with vessel density in solid tumors^16–18,35–37^; however, no significant correlation was found in the present study. On the other hand, some studies reported that angiogenesis into metastatic sites is a necessary pathway for neutrophils to infiltrate^38]– [39^. The lack of an association between blood vessels and stroma and neutrophil infiltration in the present study suggests that a new pathway of neutrophil infiltration exists in ovarian cancer, possibly via ascites. Further research is needed.

The strengths of the study include the use of ovarian cancer patient specimens and the analysis of the association between tissue and blood findings. Limitations include the small number of patients with NAC and that the chemotherapy regime varied depending on age.

In conclusion, neutrophil infiltration into disseminated lesions of the omentum has a significant impact on prognosis. The results of the present study may provide insights to generate hypotheses for refining clinical approaches.

## Electronic supplementary material

Below is the link to the electronic supplementary material.

Supplementary Material 1

Supplementary Material 2

Supplementary Material 3

Supplementary Material 4

## Data Availability

The data that support the findings of this study are available on request from the corresponding author. The data are not publicly available due to privacy or ethical restrictions.

## Declarations

### Patient and public involvement statement

Patients or the public were not involved in the design, or conduct, or reporting, or dissemination plans of our research.
