# Supplementary material for: Neutrophil infiltration in peritoneal metastasis affects prognosis in patients with ovarian cancer
Source: Sci Rep. 2025 Jul 2;15:23196. doi: 10.1038/s41598-025-05010-3 (PMC12223313; doi:10.1038/s41598-025-05010-3)
Supplement: Supplementary file 4 — Supplementary Material 4 [file 41598_2025_5010_MOESM4_ESM.docx]

**Figure legend**

Supplementary Figure 1; (A) Tables of number and rate of MPO-positive cells. Kaplan–Meier curves are shown for progression-free survival in high or low neutrophil infiltration group. (B) Forest plot of multivariate analysis for progression-free survival.

Supplementary Figure 2; (A) Correlation between number of MPO-positive cells and each neutrophil indicator or evaluation item for neutropenia. r; Pearson’s correlation coefficient. (B) Kaplan–Meier curves are shown for overall survival in each neutrophil indicator or evaluation item for neutropenia.

Supplementary Figure 3; Kaplan–Meier curves are shown for progression-free survival in vascular endothelial cell area or the number of stromal cells in peritoneal metastasis.
